# Spatial-Temporal Modelling of Disease Risk Accounting for PM2.5 Exposure in the Province of Pavia: An Area of the Po Valley

**DOI:** 10.3390/ijerph18020658

**Published:** 2021-01-14

**Authors:** Leonardo Trivelli, Paola Borrelli, Ennio Cadum, Enrico Pisoni, Simona Villani

**Affiliations:** 1 Unit of Biostatistics and Clinical Epidemiology, Department of Public Health, Experimental and Forensic Medicine, University of Pavia, 27100 Pavia, Italy; trivelli.leonardo@gmail.com (L.T.); paola.borrelli@unipv.it (P.B.); 2 Laboratory of Biostatistics, Department of Medical, Oral and Biotechnological Sciences, University “G. d’Annunzio” Chieti-Pescara, 66100 Chieti, Italy; 3 Environmental Health Unit, Agency for Health Protection, 27100 Pavia, Italy; Ennio_cadum@ats-pavia.it; 4 European Commission, Joint Research Centre (JRC), 21027 Ispra, Italy; enrico.pisoni@ec.europa.eu

**Keywords:** spatial analysis, particulate matter, cardiovascular mortality

## Abstract

Spatio-temporal Bayesian disease mapping is the branch of spatial epidemiology interested in providing valuable risk estimates in certain geographical regions using administrative areas as statistical units. The aim of the present paper is to describe spatio-temporal distribution of cardiovascular mortality in the Province of Pavia in 2010 through 2015 and assess its association with environmental pollution exposure. To produce reliable risk estimates, eight different models (hierarchical log-linear model) have been assessed: temporal parametric trend components were included together with some random effects that allowed the accounting of spatial structure of the region. The Bayesian approach allowed the borrowing information effect, including simpler model results in the more complex setting. To compare these models, Watanabe–Akaike Information Criteria (WAIC) and Leave One Out Information Criteria (LOOIC) were applied. In the modelling phase, the relationship between the disease risk and pollutants exposure (PM2.5) accounting for the urbanisation level of each geographical unit showed a strong significant effect of the pollutant exposure (OR = 1.075 and posterior probability, or PP, >0.999, equivalent to *p* < 0.001). A high-risk cluster of Cardiovascular mortality in the Lomellina subareas in the studied window was identified.

## 1. Introduction

Health effects of air pollution have continued to be investigated for a long time. The environmental exposure can affect different systems, but according to the majority of epidemiological studies, those primarily affected are the cardiovascular and the respiratory systems [1,2,3,4,5,6,7,8,9,10,11,12]. Among the air pollutants, particulate matter with aerodynamic diameter less than 2.5 µm (the so-called fine particles or PM2.5) evidenced an association with mortality for cardiovascular diseases in different epidemiological studies, as recently summarised by Franklin and co-workers [13]. The meta-analysis conducted by Hoek and co-workers [14] found an increase of 11% in cardiovascular mortality associated with a 10 μg/m^3^ increment in annual PM2.5 concentrations.

As indicated in the special report 23/2018 by the European court of auditors [15], in Europe, almost the totality of people living in urban areas were exposed to levels of air pollutants dangerous to their health, according to the World Health Organisation (WHO) [16]. Increased health risks in people living in urban areas compared with those living in rural areas were due to the fact that cities are densely populated [16]. Some studies suggested that living in the most polluted areas could be connected with an increased risk of mortality in general and in particular for cardiovascular diseases [17,18,19]. Similar evidence derived from epidemiological surveys made in Italy showed the same findings. In these cases, the impact of air pollution on mortality for cardiovascular diseases was evaluated in people living in areas characterised by having high environmental pollution due to a local facility or high level of urbanisation [20,21,22,23]. Studies investigating geographical distribution of cardiovascular mortality related to air pollutants are sparse [18,24,25] and not one concerns the Italian areas. Consequently, the present study was conducted to determine the spatio-temporal association between environmental exposure to particulate matter, PM2.5 μg/m^3^, and the risk of cardiovascular mortality in the 2010–2015 period in an Italian region with a high level of air pollution and human activities, using a Bayesian smoothed approach.

## 2. Materials and Methods

### 2.1. Study Area

**Figure 1:**
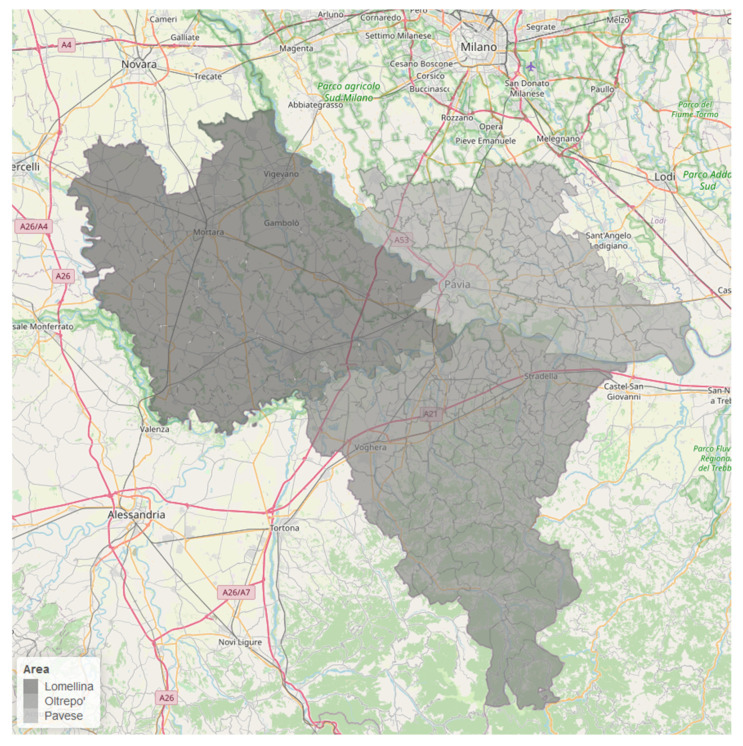
Map of study area with the three subareas.

The Province of Pavia, with a surface of 2969 km^2^, is located at 45°11′7″44 North and 09°9′45″00 East coordinates to the South of Milan, in the Lombardy Region, in the so-called Po Valley surrounded by the Alps on the Western and Northern sides and by the Apennines on the Southern side (Figure 1). According to Köppen’s climatic classification, the Po Valley is characterised by a climate ranging from humid continental to humid subtropical. The orographic conformation favours the thermic inversion in the winter period with a low mixing height from the ground to the air and many days exceeding the alert thresholds for air pollutants.

The study area was composed of 190 municipalities until 2012, when four municipalities were merged into two new administrative areas (Cornale–Bastida De’ Dossi and Corteolona–Genzone). Since then, there have been 188 municipalities. To provide comparable model results in the study period, these areas were merged from the beginning of the study (2010). The municipalities of the Province of Pavia can be divided in three subareas diversified by the geographical morphology and the type of urbanisation: the so-called Pavese (the most populated area of the Pavia Province with 202,120 inhabitants up to 1 January 2012, in 53 counties), the Lomellina (with 193,142 inhabitants, in 58 counties), and the Oltrepo’ (with 140,404 inhabitants, in 77 counties).

The Pavese is a flat area located in the Northeast part of the Province bordered to the north by the Metropolitan area of Milan, to the south by the Po river, and to the west by the Ticinum river. It includes the administrative centre of the Pavia Province (77 m above sea level), which is the most populous city in the area with its 68,352 inhabitants up to 1 January 2012. Pavia is the seat of an ancient Italian University and of numerous important Medical and Research Centres, the primary industries in the city and its hinterland. The Lomellina has always been a predominantly rural flat area located to the Northwest, wedged between the Po river and the Ticinum river. It is characterised by intensive rice farming, but there are also important industrial plants such as a refinery and a waste incinerator. There are also several small industries (i.e., shoes manufacturing and metallurgical crafts) spread around Vigevano (100 m above sea level), the second largest city of the Pavia Province (60,002 inhabits, 2012). The Oltrepo’, which literally means “the other side of the Po river”, is also a rural area located to the South and is predominantly hilly. The Apennine chain goes up to 1460 metres above sea level with Penice Mountain and this area is predominantly agricultural (viticulture).

### 2.2. The Study Design

This is an ecological study and is part of a more extensive research project named “Studio SIAMO” (Salute e Inquinamento Ambientale per il Monitoraggio dell’Oltrepò), approved by the reference Ethical Board (Fondazione Policlinico San Matteo of Pavia, protocol number 20180042126, meeting of 6 June 2018). Studio SIAMO has been developed to investigate both the environmental profile of the Broni area and health profile of the people affected by the Fibronit factory that produced asbestos-containing materials (ACM) from 1932 to 1992. The SIAMO Project has been structured in several work packages (WP), following a “funnel approach” (from the ecological level to the individual level): WP1 aimed to describe the health profile (mortality and hospital admissions) of people living in the Province of Pavia. WP2 aimed to describe the environmental profile of the Broni area and exposure assessment of people enrolled in the WP3.1. The sub-work package 3.1 (WP3.1) aimed to evaluate the long-term effect of environmental exposure to asbestos on the health of people living in Broni since 1970 (retrospective cohort design), while the sub-work package 3.2 (WP3.2) aimed to estimate the incidence of ARDs (Asbestos Related Diseases) and psychological well-being of people born in Broni between 1970 and 1980 (prospective birth cohorts design). The aim of WP4 was to evaluate the association between environmental pressure factors present in the Broni area (highlighted in WP2) and health outcomes (studied in WP3.2). Finally, the aim of WP5 was to promote awareness and set a basis for future interventions.

### 2.3. The Outcome

The outcome of interest was deaths from cardiovascular-related diseases; the International Classification of Diseases 10th revision (ICD-10) codes I00–I199 were used to identify the cases within each municipality. All deceased persons observed in the Province of Pavia between 2010 and 2015 were included in the analyses, separated by year, gender, and age.

The number of deaths related to cardiovascular diseases were taken from the mortality register of the local health agency of the Pavia province (in Italian: Agenzia di Tutela della Salute di Pavia, or ATS Pavia). 

### 2.4. Environmental Exposure

Taking into account published evidences, particulate matter 2.5 (PM2.5) was chosen to assess environmental exposure. Even if air monitoring stations belonging to the environmental network controlled by the National Environmental Agency (in Italian: Agenzia Regionale per l’Ambiente, or ARPA) were present in the Province of Pavia, stations were not spread in a uniform way, so data exposure at the municipal level were lacking. Alternatively, the modelling of the spatial pollutant concentrations might be used to determine the air pollution in areal units such as municipalities. In the present work, this second solution was adopted. PM2.5 concentrations were derived from the EMEP (European Monitoring and Evaluation Programme) the co-operative programme for monitoring and evaluation of the long-range transmission of air pollutants in Europe [26], a scientific body set up in the frame of the UNECE LRTAP (United Nation Economic Commission for Europe, Long-Range Transboundary Air Pollution convention). The data represented gridded concentrations in 2012 [27] of several air pollutants in degree (longitude–latitude) resolution from the year 2000 onwards. According to the purpose of the study, gridded data for the Pavia province were extracted in a yearly time-series from 2010 to 2015, for PM2.5. The grid was further subdivided into the 188 municipalities of the Pavia Province. For each municipality, annual mean exposure was used.

Since 2015, the National Environmental Agency (Agenzia per la Protezione dell’Ambiente, ARPA) fixed (following the requirements of the EU Air Quality Directive 2008/50 [28]) the annual limit value for the protection of human health at 25 μg/m^3^ [29].

### 2.5. The Population

The target population was those living in the Pavia Province. Open source in an ISTAT (National Institute of Statistics, in Italian: Istituto Nazionale di Statistica) portal [30] provided the gender and the age of the population resident in each municipality for each considered year. Male and female populations were distributed in 20 age classes. The population of 2012 was used as a reference in the analyses. On 1 January 2012, the overall Province population was 535,666: 258,596 males (48.28%) and 277,070 females (51.72%), according to the public data provider.

### 2.6. Degree of Urbanisation

The degree of urbanisation is a classification index (DEGURBA index) calculated by the Commission Directorates-General for Regional and Urban Policy, Agriculture and Rural Development, together with the Organisation for Economic Co-operation and Development in 2011 [31].

This urbanisation index classifies municipalities (named LAUs, or local administrative units) by the combination of geographical contiguity of LAUs and population density, based on the minimum population thresholds applied to 1 km² population grid cells. Each municipality belongs to one of these three categories: urban (densely populated areas), peri-urban (adjacent areas to urban municipalities), and rural (sparsely populated municipalities).

### 2.7. Statistical Analyses

A simple, informative (conditioning on observed frequencies in descriptive phase) Bayesian conjugate beta-binomial model was applied to assess differences between population strata (defined by the genders) for each year in the study period:

(1) $$y_{i}∼Binomial\;\left(\pi_{i},\;n_{i}\right)$$

(2) $$\pi_{i}\sim Beta\;\left(1,\;3\right)$$

where *y_i_* were the cardiovascular events of death in males by each year in the study period and *n_i_* were the total amount of deaths in the same period.

The standardised mortality rate (SMR) was defined as the ratio between observed and expected number of cases calculated by an age–gender–municipality-specific standardisation process as follows:

(3) $$Risk_{j}=\frac{\sum_{i=1}^{188}Count_{ij}}{\sum_{i=1}^{188}Population_{ij}}$$

(4) $$e_{i}=\overset{40}{\underset{j=1}{\sum }}Population_{ij}\times Risk_{j}$$

where *i* = 1, …, 188 denotes areas and *j* = 1, …, 40 denotes age–gender categories combinations. The SMR was the estimator of an unknown parameter *θ_i_* (theta *i*) obtained using a maximum likelihood estimation approach, from

(5) $$y_{i}∼Poisson\;\left(\mu_{i}\right)\;\mathrm{with}\;\mu_{i}=e_{i}\times \theta_{i}$$

where *y_i_* represents the observed counts of cardiovascular deaths in each municipality by year, *e_i_* is the expected counts of the same events for each space-time combination, and *θ_i_* is the unknown relative risk (RR). Problems related to estimates obtained using maximum likelihood estimates in case of small and weakly populated areas are well-known in the literature [32]. Typically, the spatial distribution, including weakly populated municipalities, was characterised by a high variability and did not allow to include extra-Poisson variability, typical of these kinds of studies.

To assess the spatial distribution of the disease risk, estimates provided by maximum likelihood estimators, SMR, and mortality rate were computed. Moran’s Index (I) [33], used to measure the spatial autocorrelation, was calculated, and to evaluate its statistical significance, in the frequentist meaning, a Monte Carlo simulation approach based on 10,000 simulations has been used.

Differences in risks (measured with SMRs) among the subareas (Lomellina, Pavese, and Oltrepo’) were assessed by means of the robust hierarchical pooling model. The model was:

(6) $$SMR_{\delta }∼reparametrised-Student\;T\;\left(10,\theta_{s},\;\sigma^{2}\right)$$

(7) $$\theta_{s}\sim Normal\left(\mu_{s},\;\sigma_{\theta }^{2}\right)$$

(8) $$\sigma_{\theta }^{2}\sim Inverse-scaled\;\chi^{2}\;(10,\;\mathrm{iteration})$$

where iteration ranged between 0.1 and 1.5 (100 different values), *SMR_δ_* was the estimated mean SMR by sub-area, separately by study years, *θ_s_* was the true mean of overall mean SMR, and $\sigma_{\theta }^{2}$ was the variance of the pooling effect.

To produce more reliable risk estimates, a hierarchical mixed log-linear model with a Besag, York, and Mollié (BYM) random component [34,35] in a fully Bayesian framework was applied. This model produced a clearer and more smoothed map of relative risks (RR), allowing extra-Poisson variability induced by the spatio-temporal data structure. Two Gaussian random effects composed this convolution of random components: one spatially unstructured (exchangeable or white noise in the lattice) and one spatially structured (conditional autoregressive based on a Gaussian–Markov random field in the lattice). To define neighbourhood relations between municipalities, a dichotomous adjacency sparse matrix (188 × 188 for each year in the study period) was used. In order to take into account over-dispersion included by the temporal setting of the analysis, the exchangeable random effect component including a random intercept defined by each municipality was used. Since data were sparse due to the small number of people in the majority of municipalities belonging to the Province of Pavia, the Poisson distribution was supposed for the dependent variable. The initial model including covariates was the following:

(9) $$log\left(\mu_{i}\right)=\beta_{0}+\underset{k}{\sum }\beta_{k}X_{i}+\log(e_{i})$$

(10) $$\beta_{0}\sim Normal\left(0,3^{2}\right)$$

(11) $$\beta_{k}\sim Normal\left(0,3^{2}\right)$$

where *β*_0_, the intercept of the model, represents the RR of a rural municipality exposed to the average observed pollutant hazard (that was centred), *β_k_* denotes the regression coefficient for the *k* covariates PM2.5 (centred and scaled by 15 μg/m^3^) and DEGURBA index, and *X_i_* is the observed value of each *k* covariate in the *i* municipalities. The association between the temporal parametric trend with the outcome running the same set of random components plus the scaled year (with respect to the year 2010) in the fixed component term was also assessed. For conditioning results on the known information in all models, the expected counts previously calculated were introduced as the offset of new regression.

A total of eight models differing both for the complexity of the fixed effect and the random effect component (four models with the temporal parametric trend as the fixed effect and four without the temporal parametric pattern, with different random components) were investigated (see Supplementary Materials—Models Specification). Increasing the overall complexity, the results of the previous best model (in terms of information criteria) were used to define informative priors to the following one to shrink the effect of collinearity between the spatially autocorrelated environmental exposure to the pollutant and the conditional autoregressive spatial random effect [36]. Watanabe–Akaike information criterion (or Widely–Akaike Information criteria: WAIC) [37] and Leave-One-Out Cross-Validation Information criteria (LOO-CV, or simply LOO) [38] and their asymptotically normal differences were used to identify the best model among those fitted [39,40]: information criteria were lower when the best model was chosen.

The Bayesian setting of the analysis allows to take into consideration the information between different models.

To evaluate uncertainty of regression parameters and RR estimates, posterior probabilities (PPs) [39] were computed extracting 100,000 random samples from posterior distributions, conditioning for their scales and calculating the relative frequency of the occurrence of random samples with RR higher than the pre-fixed cut-off of unitary value. In order to assess the spatial distribution of the RR for each municipality, medians of the marginal posterior distribution of fitted RRs were used. To quantify and evaluate our uncertainty around our RR estimates, PPs were calculated as the relative frequency to observe a RR higher than the pre-fixed cut-off of unitary value. A PP > 0.90 indicated that the municipality showed a higher RR estimate with respect to the provincial median RR more than 90% of the time, otherwise a PP < 0.10 implied that that municipality presented a higher RR estimate with respect to the provincial median RR less than 10%. In other words, municipalities with a PP higher than 0.90 were defined as being at “high risk”, while municipalities which presented a PP smaller than 0.10 were designated as “low risk”.

In the first modelling phase, a simple non-informative Bayesian log-linear model defined as the log expected count, without any kind of random effect, using each data point as independent, was assessed. A normal-Gaussian prior on regression parameters centred at 0 and with a standard deviation of 3 on the log OR scale, giving a plausible log OR value defined at the 95% credibility interval [−5.879, 5.875] in the parametric space, was used.

In the second modelling phase, accounting for the data structure, a random component for each municipality was introduced to take into account the over-dispersion: a non-informative Inverse-Gamma hyperprior on the variance of random component was set, while in the fixed-component, informative Normal-Gaussian priors on the log OR scale, based on the previous model results, were used.

To introduce the spatial structure of the lattice in the third modelling phase, a conditional autoregressive random component to each dichotomous neighbourhood structure was applied. The hyperprior on the variance parameter was set to be non-informative. Conditioning for the first and the second modelling phase, a set of informative priors on regression coefficients based on the second phase results was used (information criteria suggested a better fit).

In the fourth modelling phase, a BYM random component with informative distributions on hyperpriors (conditioning with previous results) and regression parameters priors was applied. No issues on lack of convergence, measured with Rhat statistics and evaluated with trace-plots, should be reported.

Hamiltonian Monte Carlo and Markov chains, or HMCMC approach [41] was used to run the entire analysis with Stan [42] and its C++ model compiler through R program [43]. From each marginal posterior distribution, 20,000 iterations were sampled from four independent Markov chains after a standard burning/warmup period, 50% of the total chain length. The convergence of each MCMC was assessed graphically with trace plots and with the Gelman-Rubin Rhat statistics [44].

To compare PM2.5 annual mean between years in the study period, analysis of variance (ANOVA) for repeated measures and its appropriate post-hoc test were used. The Bonferroni’s correction for multiple comparisons was applied.

The analyses were made using R version 3.6.1 and in particular, several R packages: rstan [45] to interface R and Stan, loo [46] to run the model comparison phase, tidyverse [47] to process raw data, tidybayes [48] to handle Stan objects in a “tidy way”, sf [49] to create spatio-temporal data-frames, spdep [50] to define and process adjacency matrices, rgdal [51] to handle Shapefiles (.shp), and ggplot2 to make maps [52] and polygons in R.

## 3. Results

### 3.1. Air Pollution and Degree of Urbanisation

Estimated PM2.5 year-municipality mean concentration from 2010 to 2012 showed an increasing pattern; in contrast, during the period 2012–2014, the distribution presented a decreasing trend, which rose again in 2015, bringing up to the initial estimated values, as shown in Table 1. The temporal trend of PM2.5 annual mean concentration was robust (*p*-value < 0.001), and all paired comparisons were significant at post-hoc test (*p*-value < 0.001) except for the comparison of 2010 and 2015.

**Figure 2:**
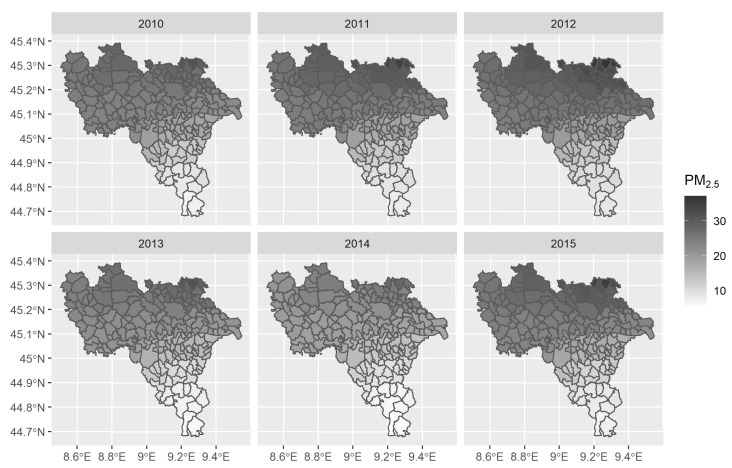
Spatio-temporal distribution of PM2.5 annual mean concentration (μg/m^3^) for each municipality: higher concentrations of particulate matter are indicated with darker colours and smaller concentrations with light colours.

The highest annual mean levels of particulate matter (≥30 μg/m^3^) were found in the municipalities of the Northern part of the Pavia province (to the border with the province of Milan, the most densely populated province of the Lombardy Region), while the lowest annual mean concentration was in the municipalities of the Southern area, known as the Oltrepo’ (≤10 μg/m^3^). This spatial pattern was the same in all the studied years, as shown in Figure 2.

**Figure 3:**
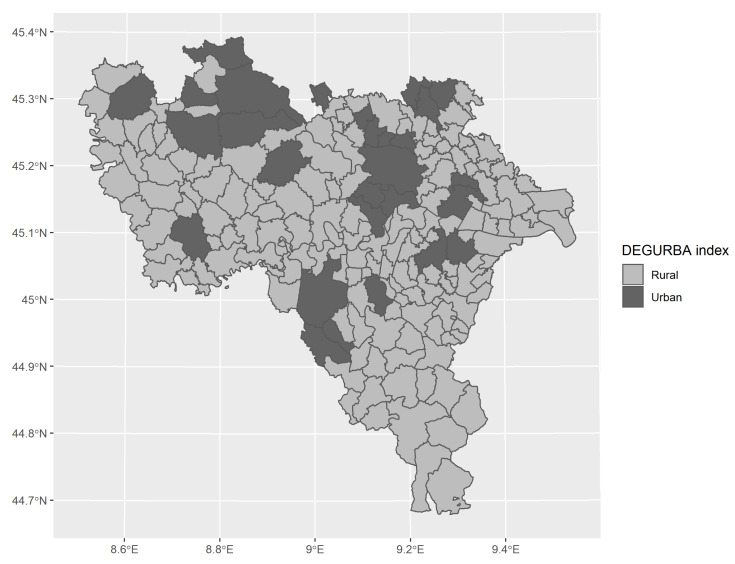
Spatial distribution of the DEGURBA index (degree of urbanisation index): the degree of urbanisation was considered invariant throughout the study period.

The spatial distribution of year-specific annual mean PM2.5 concentrations was strongly spatially auto-correlated given Moran Index (I = 0.954): Monte Carlo-simulated *p*-values were never higher than 0.001. During the study period, it was estimated that the frequency of municipalities recording total days in exceedance of daily limit value defined for PM2.5 concentration ranged from 2.12% to 57.78%, with a heterogeneous distribution across years, as reported in Table 1. The majority of municipalities (160 out of 188) was classified as rural or semi-urban by the DEGURBA index, as depicted in Figure 3, and never changed the categories in the studied years; so, the DEGURBA index was considered invariant. The municipalities classified as urban identified two clusters: the first was composed by Pavia (the most populous municipality and capital city of the province) and the hinterlands in the central part of the province, while the second included Vigevano (the most important town of Lomellina) and the surrounding municipalities, in the north-western part of the province.

### 3.2. Cardiovascular Mortality

In the overall study period, 14,183 cardiovascular deaths were observed in the study area, as summarised in Table 2. No clear trend in mortality rate was found between gender (PP > 0.05): the difference in frequencies could be considered constant during the study period.

**Figure 4:**
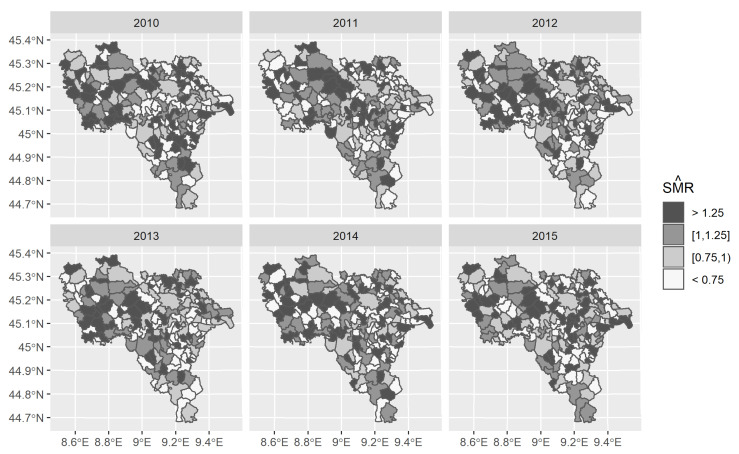
Spatio-temporal distribution of crude standardised mortality ratios (SMR) (maximum likelihood estimates from Poisson assumption).

**Figure A1:**
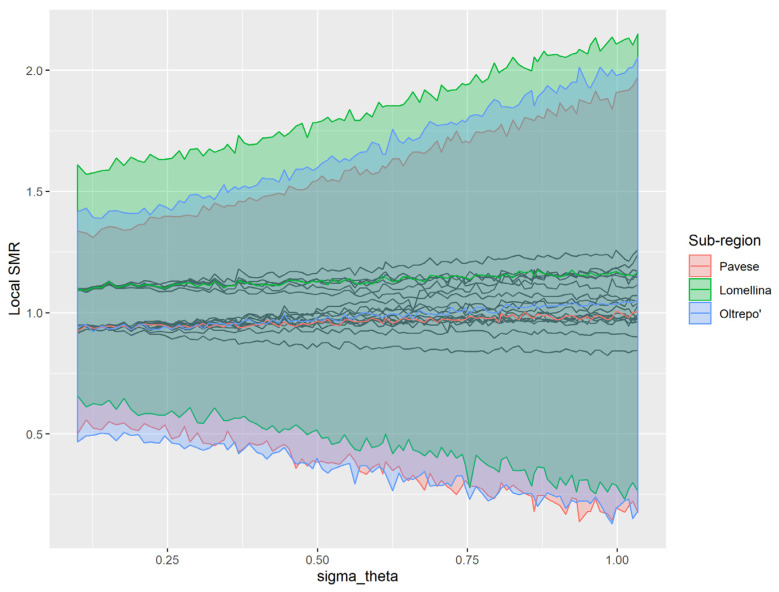
Graphical representation of the hierarchical model to compare sub-regions’ overall means with varying the standard deviation between global means Bayesian hierarchical model with 100 different hyperpriors’ specifications of variance parameters of the subareas’ mean (scaled inverse chi-squared distributions with 10 degrees of freedom and scales varying between 0.1 and 1.5, using as input the year-subareas mean) was applied. The local subareas’ estimated mean of Lomellina seemed to have different values of standardised mortality ratio (SMR) with respect to other subareas, but this difference was not significant given that the 1-PP (PP = posterior probability) values (corrected with Bonferroni’s correction) were smaller than 0.95 in the difference (p(difference>0.05).

As shown in Figure 4, the SMRs did not provide a clear spatial pattern of the disease risk estimates from 2010 to 2015. Even if higher SMRs were found in each studied year for the municipalities located in the Western part of the Pavia province (overlapping the subarea Lomellina), these excesses between Lomellina and other subareas (Pavese and Oltrepo’) were not significantly different (Figure A1, Appendix A).

Higher SMRs were always estimated for municipalities weakly populated and classified as rural areas by the DEGURBA index, apart from Pavia, which had year-specific SMRs (with its 95% Poisson exact confidence intervals) that were less than 1 per year (2010: 0.926 [0.829–1.032], 2011: 0.884 [0.787–0.989], 2012: 0.937 [0.839–1.043], 2013: 0.954 [0.853–1.063], 2014: 0.894 [0.796 1.001], 2015: 0.882 [0.790–0.983]).

### 3.3. Models

In the modelling phase, the DEGURBA index and PM2.5 concentrations were included as covariates in each model. The urban category was merged with peri-urban for the DEGURBA index, since only two municipalities were classified as urban. Whereas the PM2.5 was scaled by 15 μg/m^3^ to make the estimated parameter and the intercept meaningful, it helped the algorithm to reach the stationary state of the MCMC.

The log-linear Poisson hierarchical model (Model 2A or M2A) with a spatially unstructured random intercept for each municipality (188 random intercepts) in the study period had the best fit given the lowest information criteria (in terms of WAIC and LOOIC) for each combination of fixed effect predictors, as shown in Table 3. The best model, M2A, estimated a significant increase (PP = 0.999), 7.5%, in the risk of death for cardiovascular diseases by an increase of 15 μg/m^3^ in PM2.5, independently by urbanisation degree (Table A1, Appendix A). Also, rural municipalities showed a risk for cardiovascular diseases death 2.2% higher than urban or peri-urban ones, but in this case, the association was borderline significant (PP = 0.937). All the modelling phase results are reported in Table A2 in Appendix A.

Models without temporal trend generally performed better and were less complex than those with temporal trend when comparing LOO-CV between each model’s sub-setting for the fixed component (*p* < 0.001). Moreover, the random component of every pair of models showed a significant difference when comparing LOO-CV after Bonferroni’s correction (M2 vs M4 *p* = 0.0014, M1, M2, and M4 vs M3 *p* < 0.001, M2 vs M1 *p* < 0.001). Similar significant differences between models were found using WAICs in term of log-likelihood function (results not reported).

**Figure 5:**
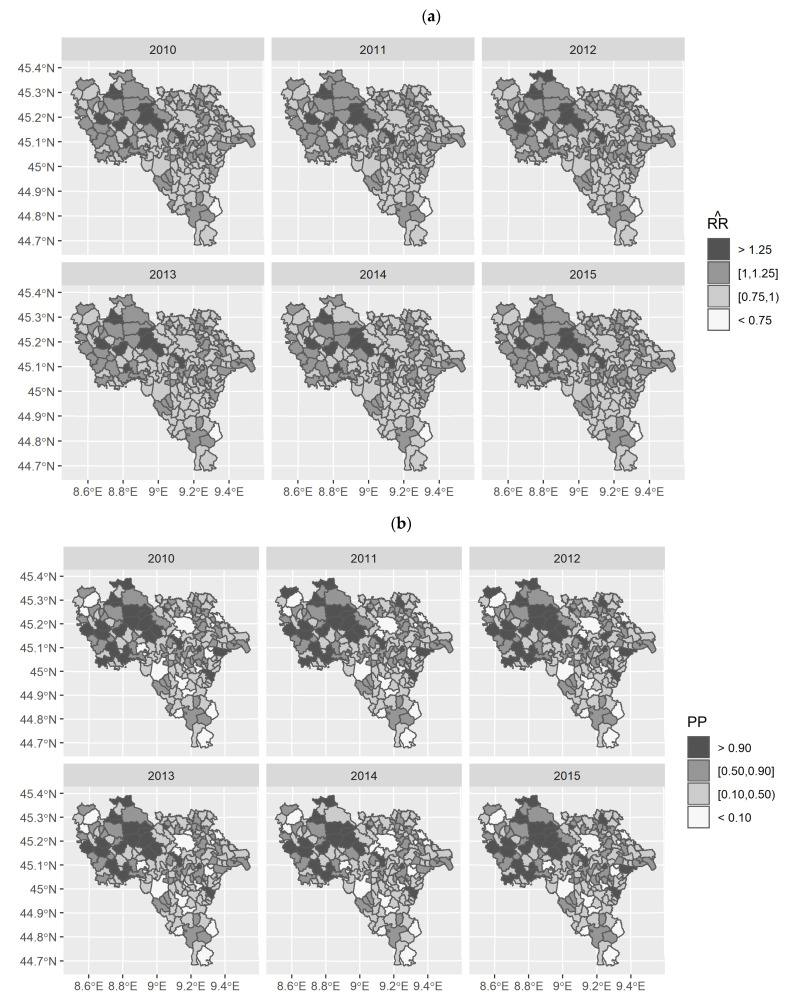
Spatio-temporal distribution of relative risk (RR) estimates provided by the Log-linear with spatially unstructured random intercept and posterior probabilities (PP). (**a**) RR estimates are “temporally” smoothed by the random intercepts posed on each municipality during the study period. Darker municipalities have a higher RR estimate, while those with whiter colours show a smaller risk estimate given by the marginal posterior distribution of RR for each area. (**b**) PP are calculated as $p\left(\hat{RR_{i}}>1\right)$. Areas marked with a darker colour are more likely to be high-risk municipalities; conversely, areas marked with a whiter colour are likely to show a smaller risk.

Based on the best model, the spatio-temporal distribution of RRs for cardiovascular diseases, controlled by PM2.5 concentrations and urbanisation degree, are presented in Figure 5a. The distribution of estimated risk of death for cardiovascular diseases did not change across the years. The presence of three clusters of high-risk for cardiovascular diseases in Lomellina in all the studied years was confirmed: 15 out 58 municipalities of this subarea showed estimated RRs at least 25% significantly (PP > 0.90) greater than the median provincial observed risk, as clearly derived comparing the spatio-temporal distribution in Figure 5a with the posterior probabilities’ distribution in Figure 5b. The largest cluster was composed by 6 municipalities placed in the central part of Lomellina showing 4 municipalities with the risk of death for cardiovascular disease more than 25% (RR > 1.25). The other clusters of RRs significantly higher than the provincial median RR were identified in the Western and Southern parts of Lomellina, neighbouring the Piedmont Region. Contrary to Lomellina, neither the Pavese nor the Oltrepo’ areas were identified as having clusters of high RRs. However, the municipalities of Arena Po, Campospinoso, and Santa Maria della Versa in Oltrepo’ showed a significantly greater risk of death for cardiovascular diseases, by 25% (RR > 1.25).

Finally, only 11 municipalities showed a risk of cardiovascular death significantly (PP < 0.10) lower with respect to the provincial median RR (Figure 5a,b) in all of the studied years and no clearly lower risk cluster was identified. Most of these municipalities were in Oltrepo’, and apart from Romagnese, the RRs for cardiovascular diseases ranged between 0.75 and 1.00. The administrative centre of the Province (Pavia) was confirmed to have a risk of death for cardiovascular disease significantly (PP < 0.10) less than the median provincial value (RR < 0.75) in all the study years.

## 4. Discussion

The cardiovascular mortality is stable in the study period and shows a heterogeneous distribution in the province of Pavia. Marked clusters of cardiovascular risk of death significantly higher than the provincial median RR were always identified in Lomellina subareas in all of the studied years, after the adjustment for air pollution level and degrees of urbanisation. Moreover, several isolated municipalities in Oltrepo’ showed RRs significantly greater than the provincial median RR.

In general, after conditioning with particulate matter and degree of urbanisation, areas with smaller PM2.5 μg/m^3^ annual mean levels had significantly smaller RRs estimates, while highly polluted areas showed significantly higher RRs. Clusters for cardiovascular mortality in Lomellina overlap the municipalities with highest PM2.5 annual concentrations, but this is not true for municipalities with elevated RRs placed in Oltrepo’, typically with the lowest air pollution levels. These findings confirm the evidence recently reported from the studies on the Seoul Metropolitan area [24] and in the Zhejiang province in China [12], in which the cardiovascular mortality is significantly related to PM10 and PM2.5, respectively. The Korean study proves that in most cases, hot spots of cardiovascular mortality are within the area characterised by high levels of particulate matter (PM10). The Chinese research finds RRs for Ischemic Heart Disease (IHD) highest in people residing in an industrial city, showing the greatest concentration levels of PM2.5 and the lowest RR in those living in a light industrial city located on a mountain or in natural reserve areas characterised by lower levels of air pollutants. However, unlike previous published studies, the municipalities with significant risk of death from cardiovascular diseases with respect to the median estimated for overall province and with elevated air pollution levels are neither industrialised places nor have high population density. So, high levels of air pollutants may be attributable to other sources common to the Po Valley. Altitude, geographical characteristics, and wind may influence the environmental pollution. An inverse relation of air pollution concentration with altitude is well-noted. In the present study, the distribution of particulate matter shows a descending gradient concentration from the Northern to the Southern municipalities: increasing the altitude, the environmental exposure seems to fall, which is confirmed in published literature [53]. The province of Pavia in the Po Valley presents stagnant wind conditions for long periods that influence the accumulation of air pollution. The Alps act as a barrier to the winds, further favouring the accumulation of pollutants. In summary, these factors contribute to the thermal inversion which creates an increase in air pollutants’ concentration in the low strata of the atmosphere.

Some municipalities in the subarea Oltrepo’ (Arena Po, Campospinoso, and Santa Maria della Versa) show a cardiovascular mortality significantly higher than the median provincial mortality. An explanation for this excess of risk is difficult to find taking into account that PM2.5 concentration in this subarea is the lowest of the Province, as shown in Figure 2, and no active source of environmental pollution is known for these municipalities: two factories were involved in the asbestos production until the total ban on use and manufacturing of asbestos from 1992 at Arena Po and at Broni, an immediate neighbour of Campospinoso [54]. Other environmental factors further PM2.5 and life habits might be responsible of this excess of risk, but these should be investigated in other studies. However, a Canadian study finds a significant association between cardiovascular mortality and exposure to concentrations of PM2.5 as low as a few micrograms per cubic metres: in this work, the estimated mean concentration of PM2.5 for subjects across the Country is 8.17 μg/m^3^ [55]. Similarly, a study conducted in the US confirms a significant association between low levels of PM2.5 (annual mean concentration range: 8–12 μg/m^3^) and death for cardiovascular diseases [56].

At the time the study was conducted and from the knowledge obtained, spatio-temporal distribution for cardiovascular mortality taking into account air pollution and degree of urbanisation had not been reported before in the open literature in the way proposed in the present paper in municipalities of this small area in the Po Valley.

The approach used in the present work to estimate the spatial distribution of mortality allows one to overcome the limits of the classical frequentist method that produces non-homogeneous maps of risk distribution (so-called “leopard spot” maps), typically affected by the random variability due to the few death events occurring in small areal units (municipalities), such as those of Oltrepo’ and Lomellina. In a map derived from the classical approach, not only it is difficult to identify clusters, but also the municipalities that show that a greater risk might not be affected by an excess of mortality. In other words, the epidemiological interpretation of frequentist maps is hard because the estimations of interest are masked by the random noise of the data and vice versa. In contrast, using a Bayesian approach the estimate of spatial distributions of risk maps is smoother and easier to interpret. The Bayesian methodology applied in the present work builds geographical distribution maps by modelling the random and the true variations separately, in a random and fixed component. In this way, the random noise from the final Bayesian map is filtered out and the map shows the true variations of cardiovascular risk of death that are interpretable as a reliable estimate of area-specific RRs. Moreover, the implemented model permits the evaluation in time of geographical distribution of cardiovascular risk of death related to levels of air pollution and degrees of urbanisation.

Finally, the information between modelling phases is borrowed in the present paper to reduce the effect of collinearity between the random clustering component and spatially autocorrelated covariates.

The Bayesian methodology maps the true distribution of the cardiovascular risk of death in the Province of Pavia. So, municipalities where RR was greater or lower than the median provincial cardiovascular risk of death indicate areas with higher or lower risk.

The main limitation of the present work is the ecological design, so the significant association of cardiovascular risk of deaths identified may be affected by ecological bias. Studies on the people living in the municipalities showing a higher risk are needed to confirm the excess described in relation to environmental factors.

## 5. Conclusions

The mortality for cardiovascular diseases in one of the most polluted Italian areas is spatially related with particulate matter adjusted by degree of urbanisation. The higher the annual mean level of PM2.5 is, the greater the relative risk for cardiovascular diseases with respect to the mortality estimated in the same area.

The presence of important industrial plants (i.e., petrochemical plant, in Sannazzaro de’ Burgundi, and waste incinerator in Vigevano and Corteolona) does not seem to be a risk factor for cardiovascular mortality for the people living in the same municipalities in which these plants are located. The cardiovascular mortality of individuals in both the areas with important industrial plants and those living in urban areas is similar. The last aspect might be related to the presence of a network of specialised medical hospitals which gives a lot of opportunities to be rapidly treated, lowering the mortality risk for cardiovascular diseases. If an ischemic attack occurs, a quick intervention is extremely important to prevent death.

Finally, in the next studies, the deprivation index should be included in the analyses to control the possible confounding effect at the individual level. In this way, the association between cardiovascular diseases and unhealthy lifestyles (i.e., alcohol and tobacco consumption) might be controlled.

The Province of Pavia falls perfectly in the field of health assessments for small areas because the study region is predominantly rural, and few deaths could result in high values of estimates provided by maximum likelihood estimators (SMR).

## Figures and Tables

**Table 1:** Summary of particulate matter (PM, annual mean) concentration in μg/m^3^ in the study period. SD = standard deviation.

	Mean	SD	Min	Max	Frequency of Municipalities Exceeding the Limit Value Defined for PM2.5 N (%)
2010	21.28	5.37	6.70	31.54	51 (27.12%)
2011	22.48	6.11	7.50	34.45	75 (39.88%)
2012	23.58	6.49	8.13	37.07	103 (57.78%),
2013	19.40	5.77	5.88	31.39	31 (16.48%)
2014	17.52	5.08	5.44	28.02	4 (2.12%)
2015	21.38	6.33	6.92	35.31	55 (29.25%)

**Table 2:** Summaries of observed cardiovascular deaths and crude mortality rate in each year of the study period by gender.

	Observed Deaths			Mortality Risk Per 1000		
	Males	Females	Overall Population	Males	Females	Overall Population
2010	963	1424	2387	3.72	5.14	4.46
2011	947	1353	2300	3.66	4.88	4.29
2012	1000	1405	2405	3.87	5.07	4.49
2013	939	1362	2301	3.63	4.92	4.30
2014	944	1316	2260	3.65	4.75	4.22
2015	1054	1476	2530	4.06	5.33	4.72

**Table 3:** Information criteria evaluated during the modelling phase. All information criteria are shown with their standard error.

Models	Without Temporal Trend (A)		With Temporal Trend (B)	
	WAIC *	LOO-CV **	WAIC	LOO-CV
M1—Log-linear	5350.4 ± 73.7	5350.4 ± 73.7	5353.2 ± 73.9	5353.3 ± 73.9
M2—Log-linear with spatially unstructured random intercept	5152.3 ± 58.7	5154.2 ± 58.8	5153.2 ± 58.8	5155.1 ± 58.8
M3—Log-linear with spatially structured random intercept	5306.4 ± 67.0	5314.5 ± 67.6	5306.9 ± 67.1	5314.6 ± 67.6
M4—Log-linear with the BYM# convolution random component	5163.8 ± 58.0	5171.3 ± 58.4	5163.7 ± 58.0	5171.2 ± 58.5

* WAIC = Watanabe—Akaike Information Criteria; ** LOO-CV = Leave-one-out Cross-Validation Information criteria; #BYM = Besag, York and Mollié.

## Data Availability

The data presented in this study are available upon request from the corresponding author. The data are not publicly available since it is property owned by the National Health Service.

## Supplementary Materials

The following are available online at https://www.mdpi.com/1660-4601/18/2/658/s1: Supplementary: Models Specification.

## Appendix A

**Table A1:** Summary statistics of marginal posterior distribution of the exponential transformation of regression parameters of our best proposed model. Posterior probabilities (PPs) can be seen as “*p*-values” complementary to 1. In the first column, there is the mean of the marginal posterior distributions (MPDs) of a parameter (OR), and in the second column, the standard deviation of that parameter. In the following 5 columns, there are the percentiles (perc.) of MPD. Finally, in the last two columns, there are the posterior probability (PP) that the estimated parameters are more, $p\left(\hat{\beta_{p}}>1\right)$, or less, $p\left(\hat{\beta_{p}}<1\right)$, than 1. A predictor was defined as a risk factor if it has a $p\left(\hat{\beta_{p}}>1\right)$ close to 1; conversely, a parameter associated with a protective factor will have $p\left(\hat{\beta_{p}}<1\right)$ close to 1.

Covariates	$e^{\hat{\beta_{p}}}$	$SD(e^{\hat{\beta_{p}}})$	2.5th perc.	25th perc.	Median	75th perc.	97.5th perc.	$p\left(\hat{\beta_{p}}>1\right)$	$p\left(\hat{\beta_{p}}<1\right)$
(Intercept)	0.977	0.009	0.958	0.970	0.977	0.984	0.997	0.013	0.986
PM2.5 in μg/m^3^	1.075	0.020	1.034	1.061	1.075	1.089	1.116	0.999	0.000
DEGURBA index rural vs urban	1.022	0.014	0.993	1.012	1.021	1.031	1.050	0.937	0.062

**Table A2:** Output of all fitted models. Posterior probabilities (PPs) can be seen as “*p*-values” complementary to 1. The first column is the mean of the marginal posterior distributions (MPDs) of a parameter (OR) and the second column is with the standard deviation of that parameter. The following 5 columns are the percentiles (perc.) of MPD. Finally, the last two columns show the posterior probability (PP) that the estimated parameters are more, $p\left(\hat{\beta_{p}}>1\right)$, or less, $p\left(\hat{\beta_{p}}<1\right)$, than 1. A predictor was defined as a risk factor if it has a $p\left(\hat{\beta_{p}}>1\right)$ close to 1; conversely, a parameter associated with a protective factor will have $p\left(\hat{\beta_{p}}<1\right)$ close to 1.

**(a) Model 1**									
					**A**				
**Covariates**	$\hat{\beta_{p}}$	$SD(\hat{\beta_{p}})$	**2.5th perc.**	**25th perc.**	**Median**	**75th perc.**	**97.5th perc.**	$p\left(\hat{\beta_{p}}>1\right)$	$p\left(\hat{\beta_{p}}<1\right)$
(Intercept)	−0.0207	0.0117	−0.0437	−0.0285	−0.0208	−0.0128	0.00221	0.0393	0.961
PM2.5 in μg/m^3^	0.0731	0.0227	0.0291	0.0577	0.0728	0.0882	0.118	0.999	0.00065
DEGURBA index rural vs urban	0.0313	0.0180	−0.00422	0.0194	0.0313	0.0436	0.0662	0.959	0.0413
					**B**				
(Intercept)	−0.0264	0.0175	−0.0609	−0.0383	−0.0263	−0.0145	0.00776	0.0644	0.936
PM2.5 in μg/m^3^	0.0747	0.0233	0.0289	0.0593	0.0746	0.0902	0.121	1.00	0.000500
DEGURBA index rural vs urban	0.0316	0.0182	−0.00454	0.0193	0.0318	0.0439	0.0670	0.959	0.0414
Calendar year	0.00210	0.00488	−0.00754	−0.00117	0.00209	0.00538	0.0117	0.671	0.329
**(b) Model 2**									
					**A**				
**Covariates**	$\hat{\beta_{p}}$	$SD(\hat{\beta_{p}})$	**2.5th perc.**	**25th perc.**	**Median**	**75th perc.**	**97.5th perc.**	$p\left(\hat{\beta_{p}}>1\right)$	$p\left(\hat{\beta_{p}}<1\right)$
Intercept	−0.0228	0.0102	−0.0426	−0.0297	−0.0228	−0.0159	−0.0026	0.0138	0.9860
PM2.5 in μg/m^3^	0.0726	0.0193	0.0341	0.0596	0.0726	0.0855	0.1100	1.0000	0.00005
DEGURBA index rural vs urban	0.0217	0.0142	−0.0063	0.0120	0.0217	0.0313	0.0494	0.9370	0.0628
					**B**				
(Intercept)	−0.0297	0.0140	−0.0573	−0.0391	−0.0298	−0.0203	−0.0023	0.0168	0.9830
PM2.5 in μg/m^3^	0.0749	0.0193	0.0368	0.0617	0.0749	0.0880	0.1130	1.0000	0.0001
DEGURBA index rural vs urban	0.0226	0.0151	−0.0069	0.0123	0.0225	0.0329	0.0517	0.9310	0.0687
Calendar year	0.0018	0.0033	−0.0046	−0.0004	0.0018	0.0040	0.0083	0.7120	0.2880
**(c) Model 3**									
					**A**				
**Covariates**	$\hat{\beta_{p}}$	$SD(\hat{\beta_{p}})$	**2.5th perc.**	**25th perc.**	**Median**	**75th perc.**	**97.5th perc.**	$p\left(\hat{\beta_{p}}>1\right)$	$p\left(\hat{\beta_{p}}<1\right)$
(Intercept)	−0.0236	0.0083	−0.0399	−0.0291	−0.0236	−0.0181	−0.0073	0.0027	0.9970
PM2.5 in μg/m^3^	0.0678	0.0201	0.0287	0.0544	0.0676	0.0814	0.1070	1.0000	0.0003
DEGURBA index rural vs urban	0.0284	0.0122	0.0046	0.0203	0.0285	0.0366	0.0522	0.9900	0.0102
					**B**				
(Intercept)	−0.0290	0.0122	−0.0529	−0.0373	−0.0289	−0.0207	−0.0050	0.0089	0.9910
PM2.5 in μg/m^3^	0.0693	0.0205	0.02910	0.0556	0.0693	0.0829	0.1090	1.0000	0.0004
DEGURBA index rural vs urban	0.0297	0.0127	0.0046	0.0211	0.0296	0.0382	0.0547	0.9890	0.0109
Calendar year	0.00144	0.00425	−0.00700	−0.00142	0.00145	0.00432	0.00974	0.634	0.366
**(d) Model 4**									
					**A**				
**Covariates**	$\hat{\beta_{p}}$	$SD(\hat{\beta_{p}})$	**2.5th perc.**	**25th perc.**	**Median**	**75th perc.**	**97.5th perc.**	$p\left(\hat{\beta_{p}}>1\right)$	$p\left(\hat{\beta_{p}}<1\right)$
(Intercept)	−0.0235	0.0103	−0.0436	−0.0304	−0.0235	−0.0166	−0.0031	0.0109	0.9890
PM2.5 in μg/m^3^	0.0768	0.0207	0.0360	0.0628	0.0769	0.0906	0.1180	1.0000	0.0002
DEGURBA index rural vs urban	0.0209	0.0140	−0.0067	0.0113	0.0207	0.0304	0.0483	0.9320	0.0680
					**B**				
(Intercept)	−0.0300	0.0141	−0.0577	−0.0395	−0.0301	−0.0206	−0.0021	0.0182	0.9820
PM2.5 in μg/m^3^	0.0792	0.0212	0.0380	0.0649	0.0793	0.09330	0.1200	1.0000	0.00005
DEGURBA index rural vs urban	0.0229	0.0150	−0.0066	0.0127	0.0229	0.03280	0.0524	0.9370	0.0632
Calendar year	0.0001	0.0044	−0.0078	−0.0020	0.0010	0.0040	0.0098	0.5880	0.4120

## References

[1] Rees J., Kanabar D., Pattani S. (2010). ABC Asthma.

[2] Yemaneberhan H., Bekele Z., Venn A., Lewis S., Parry E., Britton J. (1997). Prevalence of wheeze and asthma and relation to atopy in urban and rural Ethiopia. Lancet.

[3] D’Amato G. (1999). Outdoor air pollution in urban areas and allergic respiratory diseases. Monaldi Arch. Chest Dis..

[4] Koenig J.Q. (1999). Air pollution and asthma. J. Allergy Clin. Immunol..

[5] Künzli N., Medina S., Kaiser R., Quénel P., Horak F., Studnicka M. (2001). Assessment of deaths attributable to air pollution: Should we use risk estimates bases on time series or on cohort studies?. Am. J. Epidemiol..

[6] Baldacci S., Viegi G. (2002). Respiratory effects of environmental pollution: Epidemiological data. Monaldi Arch. Chest Dis..

[7] Pope C.A., Dockery D.W. (2006). Health effects of fine particulate air pollution: Lines that connect. J. Air Waste Manag. Assoc..

[8] Kampa M., Castanas E. (2008). Human health effects of air pollution. Environ. Pollut..

[9] Brook R.D., Rajagopalan S., Pope C.A., Brook J.R., Bhatnagar A., Diez-Roux A.V., Holguin F., Hong Y., Luepker R.V., Mittleman M.A. (2010). Particulate matter air pollution and cardiovascular disease: An update to the scientific statement from the American Heart Association. Circulation.

[10] Boldo E., Linares C., Aragonés N., Lumbreras J., Borge R., de la Paz D., Pérez-Gómez B., Fernández-Navarro P., García-Pérez J., Pollán M. (2014). Air quality modelling and mortality impact of fine particles reduction policies in Spain. Environ. Res..

[11] Khaniabadi Y.O., Goudarzi G., Daryanoosh S.M., Borgini A., Tittarelli A., De Marco A. (2017). Exposure to PM10, NO_2_, and O_3_ and impacts on human health. Environ. Sci. Pollut. Res. Int..

[12] Chen Y., Zang L., Du W., Xu D., Shen G., Zhang Q., Zou Q., Chen J., Zhao M., Yao D. (2018). Ambient air pollution of particles and gas pollutants, and the predicted health risk from long-term exposure to PM2.5 in Zhejiang province, China. Environ. Sci Pollut. Res. Int..

[13] Franklin B.A., Brook R., Pope C.A. (2015). Air pollution and cardiovascular disease. Curr. Probl. Cardiol..

[14] Hoek G., Krishnan R.M., Beelen R., Peters A., Ostro B., Brunekreef B., Kaufman J.D. (2013). Long-term air pollution exposure and cardio-respiratory mortality: A review. Environ. Health.

[15] European Court of Auditors Special Report No 23/2018: Air Pollution: Our Health Still Insufficiently Protected. https://op.europa.eu/webpub/eca/special-reports/air-quality-23-2018/en/.

[16] European Environment Agency (2017). Outdoor Air Quality in Urban Areas. https://www.eea.europa.eu/publications/environmental-indicator-report-2017.

[17] Dockery D.W., Pope C.A., Xu X., Spengler J.D., Ware J.H., Fay M.E., Ferris B.G., Speizer F.E. (1993). An Association between Air Pollution and Mortality in Six US Cities. N. Engl. J. Med..

[18] Jerrett M., Burnett R.T., Ma R., Pope C.A., Krewski D., Newbold K.B., Thurston G., Shi Y., Finkelstein N., Calle E.E. (2005). Spatial analysis of air pollution and mortality in Los Angeles. Epidemiology.

[19] Beelen R., Hoek G., van den Brandt P.A., Goldbohm R.A., Fischer P., Schouten L.J., Jerrett M., Hughes E., Armstrong B., Brunekreef B. (2008). Long-term effects of traffic related air pollution on mortality in a Dutch cohort (NLCS-AIR study). Environ. Health Perspect..

[20] Baccini M., Biggeri A., Grillo P., Consonni D., Bertazzi P.A. (2011). Health impact assessment of fine particle pollution at the regional level. Am. J. Epidemiol..

[21] Alessandrini E.R., Faustini A., Chiusolo M., Stafoggia M., Gandini M., Demaria M., Antonelli A., Arena P., Biggeri A., Canova C. (2013). Inquinamento atmosferico e mortalità in venticinque città italiane: Risultati del progetto EpiAir2. [Air pollution and mortality in twenty-five Italian cities: Results of the EpiAir2 Project]. Epidemiol. Prev..

[22] Carugno M., Consonni D., Randi G., Catelan D., Grisotto L., Bertazzi P.A., Biggeri A., Baccini M. (2016). Air pollution exposure, cause-specific deaths and hospitalizations in a highly polluted Italian region. Environ. Res..

[23] Leogrande S., Alessandrini E.R., Stafoggia M., Morabito A., Nocioni A., Ancona C., Bisceglia L., Mataloni F., Giua R., Mincuzzi A. (2019). Industrial air pollution and mortality in the Taranto area, Southern Italy: A difference-in-differences approach. Environ. Int..

[24] Lim Y.R., Bae H.J., Lim Y.H., Yu S., Kim G.B., Cho Y.S. (2014). Spatial analysis of PM10 and cardiovascular mortality in the Seul metropolitan area. Environ. Health Toxicol..

[25] Ye Z., Xu L., Zhou Z., Wu Y., Fang Y. (2018). Application of SCM with Bayesian B-Spline to Spatio-Temporal Analysis of Hypertension in China. Int. J. Environ. Res. Public Health.

[26] European Monitoring and Evaluation Programme, EMEP Programme. www.emep.int.

[27] Simpson D., Benedictow A., Berge H., Bergström R., Emberson L.D., Fagerli H., Flechard C.R., Hayman G.D., Gauss M., Jonson J.E. (2012). The EMEP MSC-W chemical transport model—technical description. Atmos. Chem. Phys..

[28] European Environment Agency Directive 2008/50/EC of the European Parliament and of the Council on Ambient Air Quality and Cleaner Air for Europe. https://www.eea.europa.eu/policy-documents/directive-2008-50-ec-of.

[29] Agenzia Regionale per la Protezione dell’Ambiente Temi Ambientali, Aria, Inquinanti, PM10 e PM2.5. https://www.arpalombardia.it/Pages/Aria/Inquinanti/PM10-PM2,5.aspx?firstlevel=Inquinanti.

[30] Demo-Geodemo Maps, Population, Demography of ISTAT. http://demo.istat.it/.

[31] European Environment Agency Degree of Urbanisation (DEGURBA). https://www.eea.europa.eu/data-and-maps/data/external/degree-of-urbanisation-degurba.

[32] Waller L., Carlin B. (2010). Bayesian Disease Mapping. Chapman Hall CRC Handb. Mod. Stat. Methods.

[33] Ripley B.D. (2005). Spatial Statistics.

[34] Besag J., York J., Mollié A. (1991). Bayesian image restoration, with two applications in spatial statistics. Ann. Inst. Stat. Math..

[35] Morris M., Wheeler-Martin K., Simpson D., Mooney S.J., Gelman A., DiMaggio C. (2019). Bayesian hierarchical spatial models: Implementing the Besag York Mollié model in Stan. Spat. Spatio-Temporal Epidemiol..

[36] Lawson A.B. (2018). Bayesian Disease Mapping: Hierarchical Modeling in Spatial Epidemiology.

[37] Watanabe S. (2010). Asymptotic equivalence of Bayes cross validation and widely applicable information criterion in singular learning theory. J. Mach. Learn Res..

[38] Gelman A., Hill J. (2007). Data Analysis Using Regression and Multilevel/ Hierarchical Models.

[39] Gelman A., Hwang J., Vehtari A. (2014). Understanding predictive information criteria for Bayesian models. Stat. Comput..

[40] Vehtari A., Gelman A., Gabry J. (2017). Practical Bayesian model evaluation using leave-one-out cross-validation and WAIC. Stat. Comput..

[41] Betancourt M. (2017). A Conceptual Introduction to Hamiltonian Monte Carlo. arXiv.

[42] Stan Development Team (2018). Stan Modeling Language Users Guide and Reference Manual, Version 2.18.0. http://mc-stan.org.

[43] R Core Team (2017). R: A Language and Environment for Statistical Computing.

[44] Gelman A., Carlin J., Stern H., Dunson D., Vehtari A., Rubin D. (2013). Bayesian Data Analysis.

[45] Stan Development Team (2018). RStan: The R Interface to Stan. R Package Version 2.17.3. http://mc-stan.org.

[46] Vehtari A., Gelman A., Gabry J. (2017). Pareto smoothed importance sampling. arXiv.

[47] Wickham H., Averick M., Bryan J., Chang W., D’Agostino-McGowan L., François R., Grolemund G., Hayes A., Henry L., Hester J. (2019). Welcome to the tidyverse. J. Open Source Softw..

[48] Kay M. (2020). Tidybayes: Tidy Data and Geoms for Bayesian Models. R Package Version 2.1.1. http://mjskay.github.io/tidybayes/.

[49] Pebesma E. (2018). Simple Features for R: Standardized Support for Spatial Vector Data. R J..

[50] Bivand R.S., Edzer P.E., Gómez-Rubio V. (2013). Applied Spatial Data Analysis with R.

[51] Bivand R. (2020). Bindings for the ‘Geospatial’ Data Abstraction Library. https://rdrr.io/cran/rgdal/.

[52] Wickham H. (2016). Ggplot2: Elegant Graphics for Data Analysis.

[53] United States Environmental Protection Agency (1978). Altitude as a factor in air pollution. National Service Center for Environmental Publications (NSCEP): EPA/600/9-78/015 (NTIS PB285645).

[54] Consonni D., DeMatteis S., Dallari B., Pesatori A.C., Riboldi L., Mensi C. (2020). Impact of an asbestos cement factory on mesothelioma incidence in a community in Italy. Environ. Res..

[55] Crouse D.L., Peters P.A., van Donkelaar A., Goldberg M.S., Villeneuve P.J., Brion O., Khan S., Odwa Atari D., Jerrett M., Pope C.A. (2012). Risk of nonaccidental and cardiovascular mortality in relation to long-term exposure to low concentrations of fine particulate matter: A Canadian national-level cohort study. Environ. Health Perspect..

[56] Hayes B.R., Lim C., Zhang Y., Cromar K., Shao Y., Reynolds R.H., Silverman T.D., Jones R.R., Park Y., Jerrett M. (2020). PM2.5 air pollution and cause-specific cardiovascular disease mortality. Int. J. Epidemiol..

