# Supplementary material for: Spatial-Temporal Modelling of Disease Risk Accounting for PM2.5 Exposure in the Province of Pavia: An Area of the Po Valley
Source: Int J Environ Res Public Health. 2021 Jan 14;18(2):658. doi: 10.3390/ijerph18020658 (PMC7828801; doi:10.3390/ijerph18020658)
Supplement: Supplementary file 1 [file ijerph-18-00658-s001.pdf]

Supplementary- model specifications

1 Model 1A - Poisson log linear model without temporal parametric trend

$$
\begin{aligned}
y_i &\sim Poisson(\mu_i)\\
\mu_i &= \theta_i \, e_i\\
log(\mu_i) &= \beta_0 + \beta_{PM2.5}PM2.5 + \beta_{Rural}I + log(e_i)\\
\beta_k &\sim Normal(0,3^2)
\end{aligned}
$$

2 Model 1B - Poisson log linear model with temporal parametric trend

$$
\begin{aligned}
y_i &\sim Poisson(\mu_i)\\
\mu_i &= \theta_i \, e_i\\
log(\mu_i) &= \beta_0 + \beta_{PM2.5}PM2.5_i + \beta_{Rural}I_i + \beta_{Year}Year_i + log(e_i)\\
\beta_k &\sim Normal(0,3^2)
\end{aligned}
$$

3. Model 2A - Poisson log linear model with county level random intercept without temporal parametric trend

$$
\begin{aligned}
y_i &\sim Poisson(\mu_i)\\
\mu_i &= \theta_i \, e_i\\
log(\mu_i) &= \beta_0 + \beta_{PM2.5}PM2.5_i + \beta_{Rural}I_i + \beta_{Year}Year_i + v_i + log(e_i)\\
\beta_0 &\sim Normal(-0.0207, 0.0117)\\
\beta_{PM2.5} &\sim Normal(0.0731, 0.0227)\\
\beta_{Rural} &\sim Normal(0.0313, 0.0180)\\
v_i &\sim Normal(0, \sigma_v^2)\\
\sigma_v^2 &\sim Inverse - Gamma(1, 0.01)
\end{aligned}
$$

4. Model 2B - Poisson log linear model with county level random intercept with temporal parametric trend

$$
\begin{aligned}
y_i &\sim Poisson(\mu_i)\\
\mu_i &= \theta_i \, e_i\\
log(\mu_i) &= \beta_0 + \beta_{PM2.5}PM2.5_i + \beta_{Rural}I_i + \beta_{Year}Year_i + v_i + log(e_i)\\
\beta_0 &\sim Normal(-0.0264, 0.0175)\\
\beta_{PM2.5} &\sim Normal(0.0747, 0.0233)
\end{aligned}
$$

$$\beta_{Rural} \sim Normal(0.0316, 0.0182)$$

$$\beta_{Year} \sim Normal(0.00210, 0.00488)$$

$$v_i \sim Normal(0, \sigma_v^2)$$

$$\sigma_v^2 \sim Inverse - Gamma(1, 0.01)$$

5. Model 3A - Poisson log linear model with county level spatial random intercept without temporal parametric trend

$$y_i \sim Poisson(\mu_i)$$

$$\mu_i = \theta_i \, e_i$$

$$log(\mu_i) = \beta_0 + \beta_{PM2.5}PM2.5_i + \beta_{Rural}I_i + \beta_{Year}Year_i + u_i + log(e_i)$$

$$\beta_0 \sim Normal(-0.0207, 0.0117)$$

$$\beta_{PM2.5} \sim Normal(0.0731, 0.0227)$$

$$\beta_{Rural} \sim Normal(0.0313, 0.0180)$$

$$u_i|u_{j\neq i} \sim Normal\left(\frac{\sum_{j\neq i} c_{ij}u_i}{\sum_{j\neq i} c_{ij}}, \frac{\sigma_u^2}{\sum_{j\neq i} c_{ij}}\right)$$

$$\sigma_u^2 \sim Inverse - Gamma(1, 0.01)$$

6. Model 3B - Poisson log linear model with county level spatial random intercept with temporal parametric trend

$$y_i \sim Poisson(\mu_i)$$

$$\mu_i = \theta_i \, e_i$$

$$log(\mu_i) = \beta_0 + \beta_{PM2.5}PM2.5_i + \beta_{Rural}I_i + \beta_{Year}Year_i + u_i + log(e_i)$$

$$\beta_0 \sim Normal(-0.0264, 0.0175)$$

$$\beta_{PM2.5} \sim Normal(0.0747, 0.0233)$$

$$\beta_{Rural} \sim Normal(0.0316, 0.0182)$$

$$u_i|u_{j\neq i} \sim Normal\left(\frac{\sum_{j\neq i} c_{ij}u_i}{\sum_{j\neq i} c_{ij}}, \frac{\sigma_u^2}{\sum_{j\neq i} c_{ij}}\right)$$

$$\sigma_u^2 \sim Inverse - Gamma(1, 0.01)$$

7. Model 4A - Poisson log linear model with county level spatial and non-spatial random intercept without temporal parametric trend

$$y_i \sim Poisson(\mu_i)$$

$$\mu_i \ = \ \theta_i \, e_i$$

$$log(\mu_i) = \ \beta_0 + \beta_{PM2.5} PM2.5_i + \beta_{Rural} I_i + \beta_{Year} Year_i + u_i + v_i + log(e_i)$$

$$\beta_0 \ \sim \ Normal(-0.0207, 0.0117)$$

$$\beta_{PM2.5} \ \sim \ Normal(0.0731, 0.0227)$$

$$\beta_{Rural} \ \sim \ Normal(0.0313, 0.0180)$$

$$u_i | u_{j \neq i} \ \sim \ Normal\left(\frac{\sum_{j \neq i} c_{ij} u_i}{\sum_{j \neq i} c_{ij}}, \frac{\sigma_u^2}{\sum_{j \neq i} c_{ij}}\right)$$

$$v_i \ \sim \ Normal(0, \sigma_v^2)$$

$$\sigma_u^2 \ \sim \ Inverse - Gamma(6.3462, 0.1280)$$

$$\sigma_v^2 \ \sim \ Inverse - Gamma(22.8692, 0.6121)$$

8. Model 4B - Poisson log linear model with county level spatial and non-spatial random intercept without temporal parametric trend

$$y_i \ \sim \ Poisson(\mu_i)$$

$$\mu_i \ = \ \theta_i \, e_i$$

$$log(\mu_i) = \ \beta_0 + \beta_{PM2.5} PM2.5_i + \beta_{Rural} I_i + \beta_{Year} Year_i + u_i + v_i + log(e_i)$$

$$\beta_0 \ \sim \ Normal(-0.0264, 0.0175)$$

$$\beta_{PM2.5} \ \sim \ Normal(0.0747, 0.0233)$$

$$\beta_{Rural} \ \sim \ Normal(0.0316, 0.0182)$$

$$\beta_{Year} \ \sim \ Normal(0.00210, 0.00488)$$

$$u_i | u_{j \neq i} \ \sim \ Normal\left(\frac{\sum_{j \neq i} c_{ij} u_i}{\sum_{j \neq i} c_{ij}}, \frac{\sigma_u^2}{\sum_{j \neq i} c_{ij}}\right)$$

$$v_i \ \sim \ Normal(0, \sigma_v^2)$$

$$\sigma_u^2 \ \sim \ Inverse - Gamma(6.3361, 0.1262)$$

$$\sigma_v^2 \ \sim \ Inverse - Gamma(22.8458, 0.6145)$$
